# Beta-blockers in refractory hypoxemia on venovenous extracorporeal membrane oxygenation: a double-edged sword

**DOI:** 10.1186/s13054-023-04648-7

**Published:** 2023-09-20

**Authors:** Dawid L. Staudacher, Tobias Wengenmayer, Matthieu Schmidt

**Affiliations:** 1 Interdisciplinary Medical Intensive Care, Faculty of Medicine and Medical Center, University of Freiburg, Hugstetterstrasse 55, 79106 Freiburg, Germany; 2 1166-ICAN, Institute of Cardiometabolism and Nutrition, APHP, Hôpital Pitié- Salpêtrière, Service de Médecine Intensive-Réanimation, Institut de Cardiologie, Sorbonne Université, Paris, France

Up to 10% of patients admitted to the intensive care unit (ICU) suffer from acute respiratory distress syndrome (ARDS) [1]. Severe respiratory failure can result in refractory hypoxemia, characterized by diminished arterial oxygen content and subsequent tissue hypoxia [2]. To address hypoxia, venovenous extracorporeal membrane oxygenation (V-V ECMO) can be employed, delivering up to 6–7 L per minute of fully oxygenated blood to the venous circulation [2, 3].

Despite V-V ECMO, persistent hypoxemia may occur [4]. The primary cause of hypoxemia is often limited pump preload, leading to reduced ECMO circuit blood flow (Q_ECMO_) [5]. If hypoxemia persists despite increased Q_ECMO_, adequate hemoglobin levels, and minimized recirculation have to be ensured. If all these rescue maneuvers failed, some reports propose the utilization of beta-blockers for refractory hypoxemia despite adequate Q_ECMO_ [4, 6, 7]. However, physiologically, this approach might yield counterintuitive effects as beta-blockers can decrease tissue oxygenation despite raising arterial oxygen saturation (SaO_2_).

We aim to emphasize potential risks associated with using beta-blockers for refractory hypoxemia during V-V ECMO. To begin, the oxygen content in the arterial blood can be estimated by the following formula [8]: arterial oxygen content (CaO_2_) = (SaO_2_ × Hb × 1.34) + (PaO_2_ × 0.003), where CaO_2_ represents the arterial oxygen content [mL/dL], SaO_2_ represents the arterial oxygen saturation [%], Hb represents the concentration of hemoglobin [g/dL], PaO_2_ represents the partial pressure of oxygen in arterial blood [mmHg] and 0.003 is a constant that accounts for the small amount of oxygen dissolved in the plasma. The oxygen present in arterial blood is subsequently conveyed to the tissues by the circulation.

Arterial oxygen delivery (DO_2_) can be therefore estimated by the following formula: DO_2_ = CaO_2_ × CO, where CO [L/min] represents cardiac output (CO). DO_2_ can therefore be improved by increasing either SaO_2_, hemoglobin levels, or CO. In V-V ECMO, oxygenated blood from the ECMO mixes with deoxygenated blood from the venous circulation thereby increasing SaO_2_. In most patients with pulmonary failure, Q_ECMO_ is lower than CO, still providing arterial oxygen saturation up to 100%. Maintaining Q_ECMO_/CO > 0.6 is one of the objectives of physicians as it seems to be associated with adequate oxygenation on V-V ECMO [9]. In hyperdynamic circulatory states such as sepsis, CO significantly surpasses maximum Q_ECMO_. When Q_ECMO_/CO is < 0.6, too much-deoxygenated blood from the circulation mixes with the oxygenated blood returned from the ECMO, resulting in a decrease in SaO_2_. Therefore, the Q_ECMO_/CO ratio is of paramount importance to properly oxygenate arterial blood. In the rare scenario of Q_ECMO_/CO < 0.6 at maximum Q_ECMO_, decreasing CO to increase the Q_ECMO_/CO ratio might appeal as a viable therapeutic target.

Some studies have shown an increase in SaO_2_ by beta-blocker therapy in severely hypoxemic ECMO patients [6, 7]. However, reducing CO by beta-blocker therapy will increase SaO_2_ at the cost of a reduction in DO_2_. Since tissue oxygenation ultimately depends on DO_2_, beta-blocker therapy can aggravate tissue hypoxia, see Fig. 1a. We tested this hypothesis in three mildly hypoxic patients in our ICU using continuous metoprolol infusion (dose 8.7 ± 1.2 mg/h). The average age was 45.5 ± 5.2 years, and the indication for V-V ECMO was ARDS in all patients. Q_ECMO_ and Q_ECMO_/CO ratio at baseline were 4.3 ± 0.5 l/min and 0.5 ± 0.1, respectively. Beta-blockers increased the Q_ECMO_/CO ratio (0.7 ± 0.1) at the cost of a decrease of CO but also importantly DO_2_, see Fig. 1b.

**Fig. 1:**
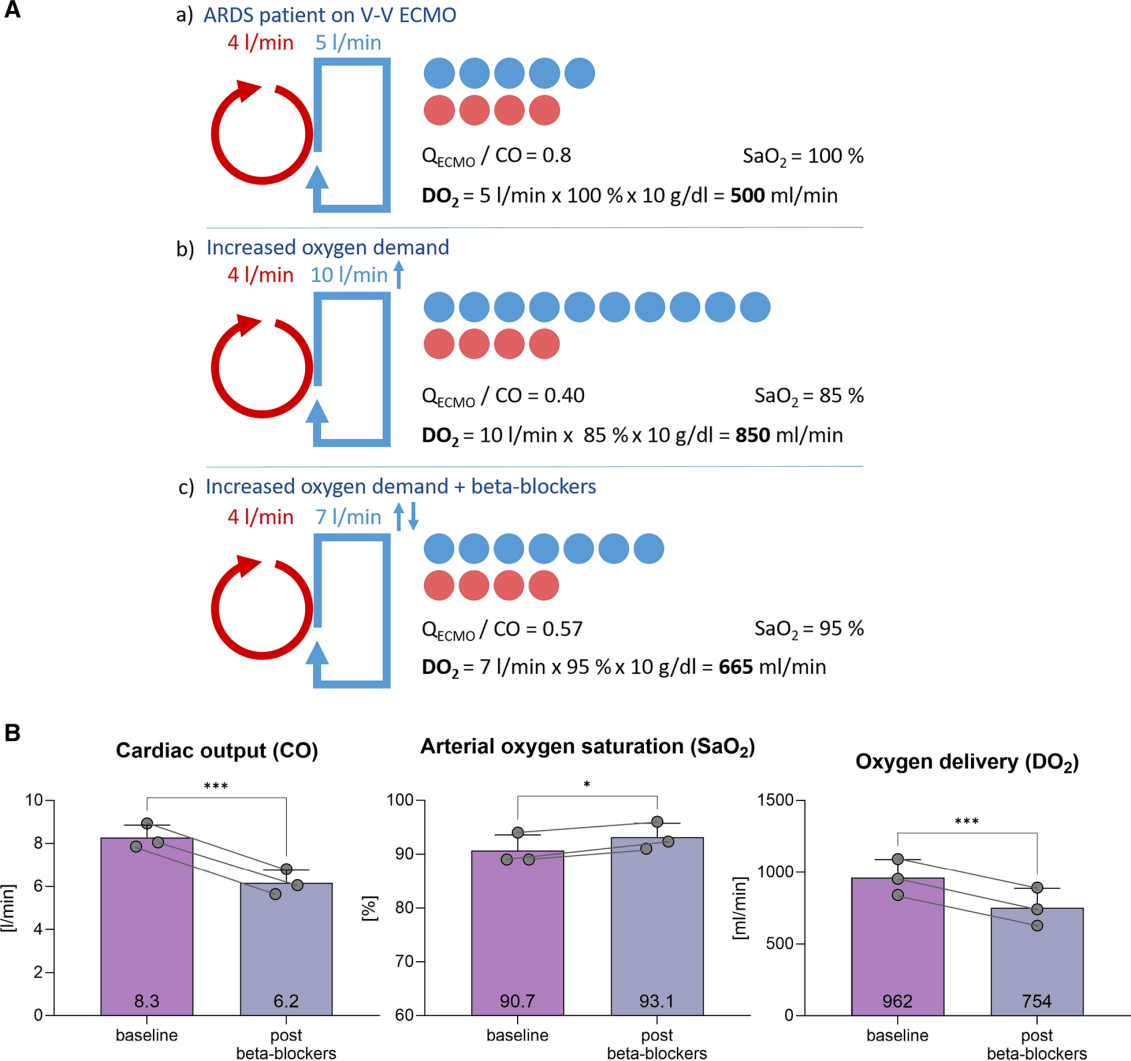
**A** Schematic representation of ECMO flow and cardiac output. Red indicates V-V ECMO flow, and blue indicates cardiac output. For illustrative purposes, recirculation is neglected; **a** Patient with ARDS and V-V ECMO support. The Q_ECMO_/CO ratio is 0.80, with saturation at 100%. DO_2_ is 500 ml/min. **b** The same patient with increased oxygen demand, for example, due to infection and fever. Q_ECMO_ remains the same while CO is increased. This results in a ratio of 0.40, saturation of 85%, but a significantly increased DO_2_ of 850 ml/min. **c** Patient with increased oxygen demand treated with beta-blocker. The higher Q_ECMO_/CO ratio improved arterial oxygen saturation, but the DO_2_ drops to 665 ml/min. **B** Displays three ARDS patients undergoing V-V ECMO therapy, in whom beta-blockers were titrated based on their effects. The measurements were taken three times each after reaching a steady state

DO_2,_ oxygen extraction and anaerobic glycolysis (increasing lactate levels) should be monitored closely when beta-blockers are used for refractory hypoxemia on V-V ECMO. Beta-blockers might only be advisable in situations where cardiac output is increased inadequately and not driven by oxygen demand. Targeting mild hypothermia, analgesic treatment, and increased sedative are some examples of simple measures that should be done as first-line treatment to improve Q_ECMO_/CO ratio. Beta-blockers should be regarded as therapy for very rare cases, considered only when an increased CO secondary to an increased oxygen demand is excluded.

## Data Availability

On request available.

## Abbreviations

CaO_2_: Arterial oxygen content
CO: Cardiac output
DO_2_: Oxygen delivery
Hb: Hemoglobin (g/dL)
PaO_2_: Partial pressure of oxygen in arterial blood [mmHg]
Q_ECMO_: Venovenous extracorporeal membrane oxygenation blood flow
SaO_2_: Arterial oxygen saturation
V-V ECMO: Venovenous extracorporeal membrane oxygenation
