# Correction: Beta-blockers in refractory hypoxemia on venovenous extracorporeal membrane oxygenation: a double-edged sword

**DOI:** 10.1186/s13054-024-05242-1

**Published:** 2025-02-03

**Authors:** Dawid L. Staudacher, Tobias Wengenmayer, Matthieu Schmidt

**Affiliations:** 1 Interdisciplinary Medical Intensive Care, Faculty of Medicine and Medical Center, University of Freiburg, Hugstetterstrasse 55, 79106 Freiburg, Germany; 2 1166‑ICAN, Institute of Cardiometabolism and Nutrition, APHP, Hopital Pitie‑ Salpetriere, Service de Medecine Intensive‑Reanimation, Institut de Cardiologie, Sorbonne Universite, Paris, France

**Correction: Critical Care (2023) 27:360** 10.1186/s13054-023-04648-7

Following publication of the original article [1], the authors identified an errors in Figure 1. It should indicate CO = 0.80 instead of CO = 0.67 in A a) ARPDS patient on V-V ECMO and g/dl instead of mg/dl in A a), b) and c). Both the incorrect and correct Figure 1 are given hereafter.

The incorrect Figure 1:

**Fig. 1:**
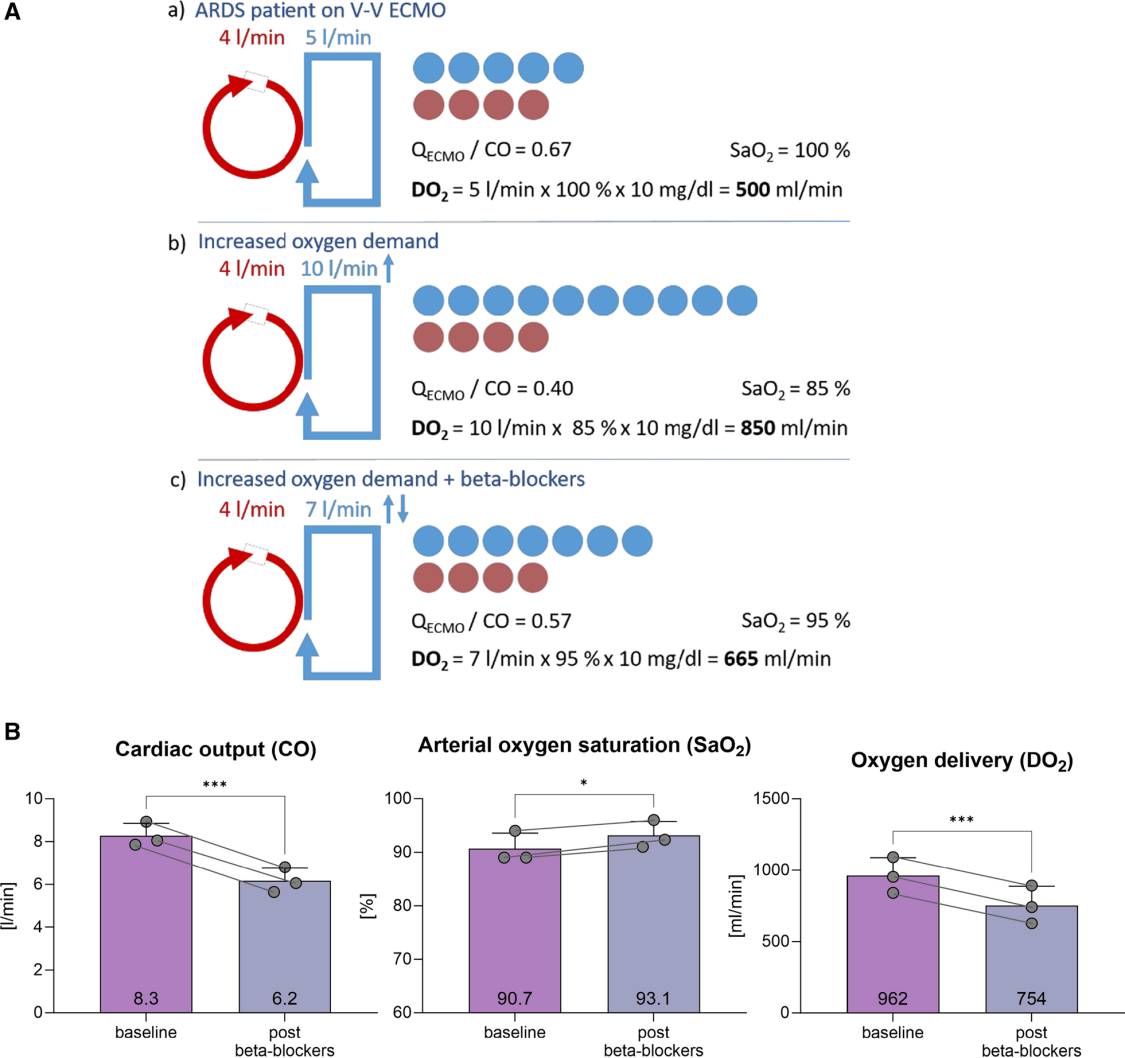
**A** Schematic representation of ECMO flow and cardiac output. Red indicates V-V ECMO flow, and blue indicates cardiac output. For illustrative purposes, recirculation is neglected; **a** Patient with ARDS and V-V ECMO support. The Q_ECMO_/CO ratio is 0.67, with saturation at 100%. DO_2_ is 500 ml/min. **b** The same patient with increased oxygen demand, for example, due to infection and fever. Q_ECMO_ remains the same while CO is increased. This results in a ratio of 0.40, saturation of 85%, but a significantly increased DO2 of 850 ml/min. **c** Patient with increased oxygen demand treated with beta-blocker. The higher Q_ECMO_/CO ratio improved arterial oxygen saturation, but the DO2 drops to 665 ml/min.** B **Displays three ARDS patients undergoing V-V ECMO therapy, in whom beta-blockers were titrated based on their effects. The measurements were taken three times each after reaching a steady state

The correct Figure 1:

**Fig. 1:**
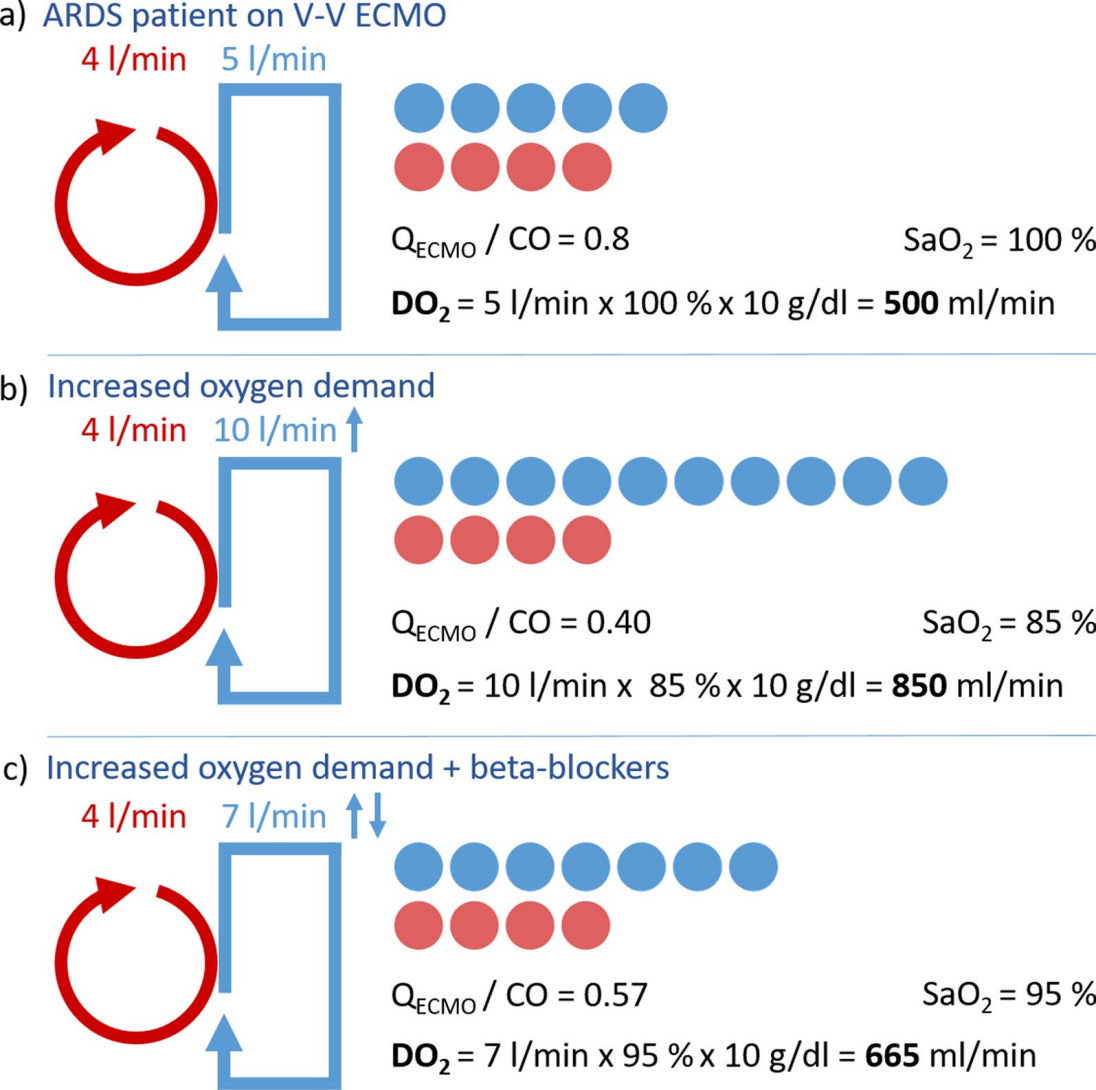
**A** Schematic representation of ECMO flow and cardiac output. Red indicates V-V ECMO flow, and blue indicates cardiac output. For illustrative purposes, recirculation is neglected; **a** Patient with ARDS and V-V ECMO support. The Q_ECMO_/CO ratio is 0.80, with saturation at 100%. DO_2_ is 500 ml/min. **b** The same patient with increased oxygen demand, for example, due to infection and fever. Q_ECMO_ remains the same while CO is increased. This results in a ratio of 0.40, saturation of 85%, but a significantly increased DO2 of 850 ml/min. **c** Patient with increased oxygen demand treated with beta-blocker. The higher Q_ECMO_/CO ratio improved arterial oxygen saturation, but the DO2 drops to 665 ml/min.** B **Displays three ARDS patients undergoing V-V ECMO therapy, in whom beta-blockers were titrated based on their effects. The measurements were taken three times each after reaching a steady state

Figure 1 has been updated in this correction article and the original article [1] has been corrected.
